# Is triple antithrombotic therapy a safe option in patients with AF who receive drug-eluting stents?: a review article

**DOI:** 10.1186/s43044-023-00402-0

**Published:** 2023-08-28

**Authors:** Ali Alshahrani, Sean O’Nunain

**Affiliations:** 1 Faculty of Biology, Medicine and Health, School of Health Sciences, University of Manchester, Oxford Road, Greater Manchester, UK; 2 Department of Invasive Cardiovascular Technology, King Saud bin Abdulaziz University for Health Sciences, Riyadh, Kingdom of Saudi Arabia; 3 King Abdulaziz Medical City, Riyadh, Kingdom of Saudi Arabia; 4 Brighton and Sussex Medical School, University of Brighton and University of Sussex, Falmer, Brighton, UK

**Keywords:** Atrial fibrillation, Percutaneous coronary intervention, Triple antithrombotic therapy, Safety

## Abstract

**Background:**

Optimal antithrombotic therapy depicts a challenge to clinicians treating atrial fibrillation (AF) patients who are undergoing percutaneous coronary intervention (PCI). Theoretically, these patients would require a combination therapy of oral anticoagulant and dual antiplatelet therapy (DAPT) with aspirin and a P2Y12 inhibitor, known as triple antithrombotic therapy (TAT). However, TAT is known to carry a significant risk of bleeding. The purpose of the present paper is to provide a focused review of the evidence about the safety of TAT as well as to address contemporary directions regarding antithrombotic therapy following PCI in patients with AF who received a drug-eluting stent.

**Main body:**

Novel oral anticoagulant studies consistently demonstrated a better safety profile when compared to Vitamin K antagonist (warfarin), especially in AF patients who have other indications of DAPT after PCI. Evidence from several studies showed that the use of TAT in AF patients undergoing stent implantation or PCI has no significant clinical benefit with more risk of major bleeding when compared to DAT. Therefore, the current recommendations for AF have taken into account the mounting evidence of antithrombotic treatment after PCI in AF patients, which has caused a major shift away from the TAT strategy toward DAT over time.

**Conclusions:**

Cardiologists face challenges in determining the best antithrombotic treatment for AF patients after PCI with DES implantation. Growing data suggest that TAT is associated with considerable bleeding and worse safety, without significant effectiveness. Hence, TAT is strictly applied for individuals with significant thrombotic risk and low bleeding risk, and for a limited duration. This paper highlights the safety concerns of TAT and current trends in antithrombotic therapy after PCI in patients with AF and DES.

## Background

Patients undergoing percutaneous coronary intervention (PCI) occasionally have or acquire accompanying indications for anticoagulation. These can include atrial fibrillation (AF), deep vein thrombosis (DVT), and mechanical valve replacements. Among these, AF is the most frequent concomitant indication for anticoagulant therapy [1]. AF is the most common sustained arrhythmia, causing numerous complications associated with increased mortality and morbidity. Studies have shown that AF prevalence is strongly age-dependent, with an exponential prevalence of AF being seen in relation to the increased average age in the population. This arrhythmia can happen because of variable pathophysiological mechanisms in the atria, which cause a loss of organized atrial contractility and shortness of atrial refractoriness. Factors contributing to the arrhythmogenic substrate include aging, activation of neurohormones, and chronic atrial dilatation secondary to structural heart disease. All of these can generate histological alterations in the atria, leading eventually to tissue fibrosis and atrial remodeling. In those predisposed to AF, ectopic beats originating from the pulmonary veins trigger sustained arrhythmia [2].

Consequently, these irregular heartbeats caused by AF may increase the likelihood of clot formation within the atria, which can eventually embolize and cause stroke and other ischemic or thrombotic events. Therefore, anticoagulant therapy has become a mainstay of the management of AF, by minimizing thromboembolic events in those at high risk [3]. The main risk factors for stroke are included in the clinical CHA2DS2-VASc score (Table 1), which is used to predict thromboembolic risk (stroke) in AF patients. International guidelines recommend (IA) starting oral anticoagulant (OAC) therapy in AF patients with a CHA_2_DS_2_-VASc score ≥ 2 (in males) and ≥ 3 (in females) for reducing stroke risk [4, 5]. On the other hand, the HAS-BLED scores were developed for the risk stratification of bleeding risk, since these anticoagulants can lead to unwanted bleeding effects. This score can allow early recognition of patients with high bleeding risk (HAS-BLED score ≥ 3). Some of the factors within this score are modifiable factors that clinicians can address during the management of AF patients [6]. (Table 1).

**Table 1:** (2020 ESC Guidelines) for the diagnosis and management of AF

CHA2DS2-VASc	Score	HAS-BLED	Score
CHF (LVEF < 40%)	1	Hypertension > 160mmHg systolic	1
Hypertension	1	Abnormal renal or liver function	1 or 2
Age ≥ 75 y	2	Stroke	1
Diabetes	1	Previous history of major Bleeding	1
Stroke	2	Labile INR	1
Vascular disease	1	Elderly (Age > 65)	1
Age 65–74	1	Drug or alcohol use—NSAIDs, antiplatelet agents, alcohol > 8 weeks/week	1 or 2
Female sex	1		

Non-sex CHA_2_DS_2_-VASc score of ≥ 2 warrants anticoagulation therapy, while patients with HAS-BLED of ≥ 3 are at higher risk of bleeding. Please note that there are some modifiable risk factors in both scores which clinicians should consider during the management of AF patients and risk balancing between ischemic and embolic risks

**Fig. 1:**
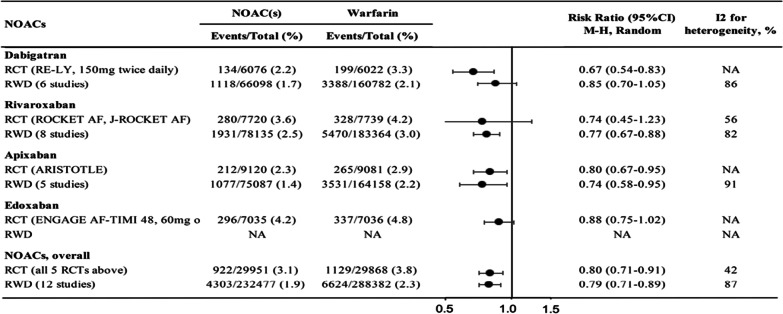
Evidence collated during the process of NOACs inclusion into the 21st WHO Model List of Essential Medicines: this table shows data from RCTs and reporting real world data (RWD) about the efficacy of NOACs versus warfarin in reducing stroke in non-valvular AF patients. NOACs were associated with either similar or superior outcomes at preventing stroke event. DOI: http://doi.org/10.5334/gh.608

**Fig. 2:**
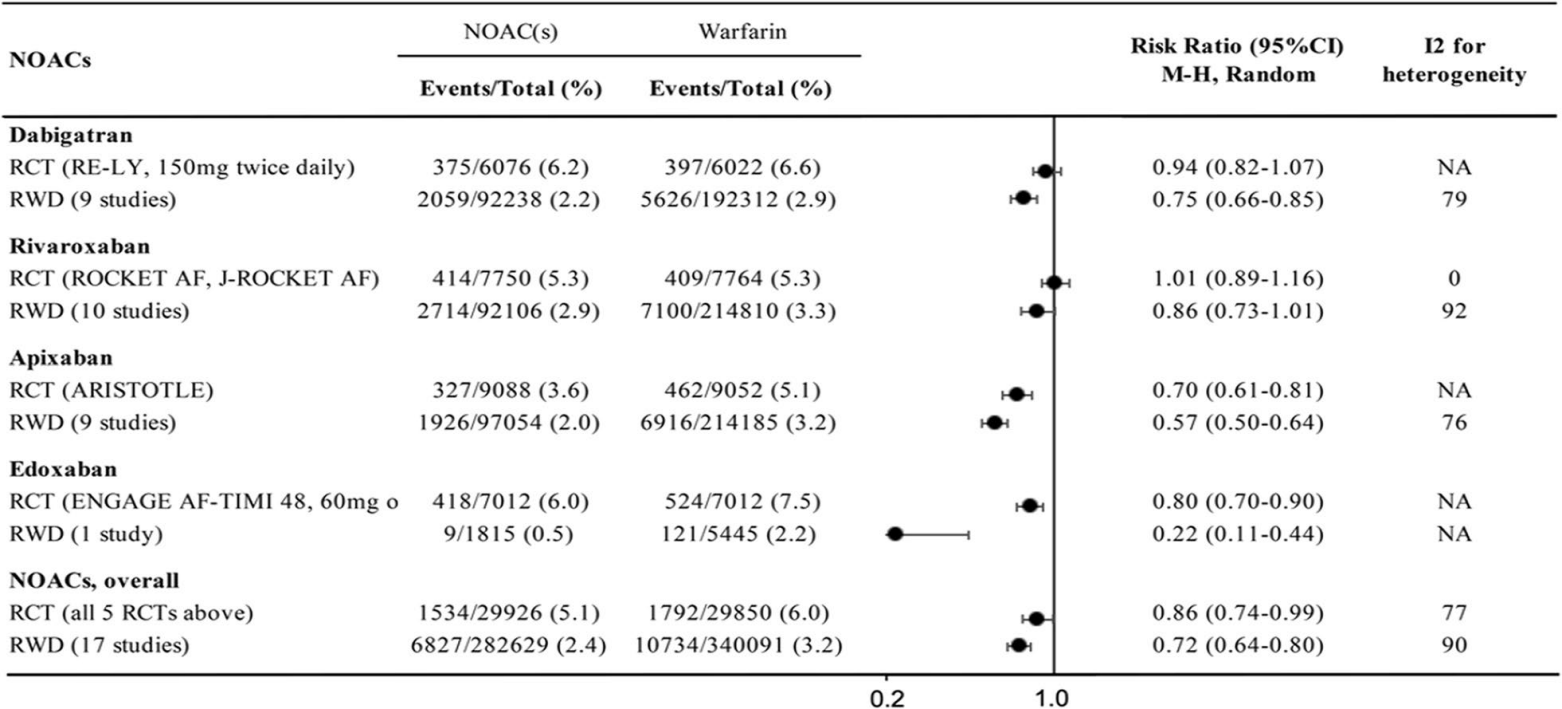
Evidence collated during the process of NOACs inclusion into the 21st WHO Model List of Essential Medicines: this table shows the risks of major bleeding in non-valvular AF patients treated by NOACs versus warfarin in non-valvular AF patients. As overall NOACs had lower major bleeding and intracranial hemorrhage (ICH) in comparison with warfarin. DOI: http://doi.org/10.5334/gh.608

For decades, the only available OAC as a primary therapy was warfarin, a vitamin K antagonist (VKA). More recently, newer medications called novel oral anticoagulants (NOACs) or direct oral anticoagulants (DOACs) have been introduced as alternatives to warfarin after having demonstrated their efficacy and safety in several randomized controlled clinical trials (RE-LY [2009] [7], ARISTOTLE [2011] [8], and ROCKET AF [2011] [9]) [10]. Moreover, antidotes are now available for NOACs, unlike a few years ago, when many physicians were wary of prescribing these medications as they were concerned about emergency surgery being required subsequently. Reasonable evidence showed that idarucizumab can be used for the reversal of dabigatran and andexanet alfa as an antidote for rivaroxaban and apixaban. This led the American College of Cardiology/American Heart Association to release an update on the current guidelines of AF management and spontaneous intracranial cerebral hemorrhage (ICH) [11]. Given the many choices now available for patients who require anticoagulation, clinicians should consider the benefits versus risks of each anticoagulant before administration. The current international guidelines prefer NOACs over warfarin for stroke prevention in AF whenever suitable to the patient’s condition. This evolution in guidelines comes after NOACs showed better safety outcomes besides other advantages such as faster onset, few drug interactions, and less bleeding. The general comparison “non-PCI context” of each anticoagulant is beyond the scope of this paper. However, for better consideration, summary tables are provided below to show the efficacy and safety of NOACs versus warfarin (see Figs. 1 and 2) [12].

The incidence of AF in patients with coronary artery disease (CAD) is estimated to be about 20%. Therefore, a number of AF patients with CAD (= 1 score in CHA2DS2-VASc) will require PCI. Dual antiplatelet therapy (DAPT) is usually prescribed after drug-eluting stent (DES) implantation for a period of 12 months as per international guidelines [13, 14]. DAPT includes aspirin (cyclo-oxygenase-1 inhibitor), and platelet adenosine diphosphate P2Y12 receptor inhibitors (e.g., clopidogrel, prasugrel, and ticagrelor) [15]. It is undeniable that this group of patients appears clinically challenging, because they continue to be at high risk of thrombosis due to both AF and the implanted DES; thus, they require adequate protection from both processes. Indeed, the pathophysiology of thrombus formation during AF (discussed above) is different from the clotting within the stent which is mainly due to high shear stress caused by stent under-expansion and the activation of platelets [16, 17].

Patients with AF and CAD will most commonly have an indication for anticoagulant (warfarin or NOACs) as well as DAPT. These combined medications are known as triple antithrombotic therapy (TAT). Unfortunately, such a combination has an increased risk of bleeding (two- to three-fold) when compared to DAPT alone [18]. Moreover, it necessitates case-by-case clinical judgment to weigh and balance thromboembolic “ischemic” risk versus bleeding risk [15]. It has been reported that patients with myocardial infarction who were given numerous antithrombotic therapies had an increase in hospital admission for bleeding. In particular, the incidence of bleeding was significantly increased in individuals who received triple treatment compared to those who received DAPT or monotherapy [19]. In the section that follow, the safety of TAT in AF patients following PCI will be critically discussed based on the available evidence from pivotal trials, and it will be compared to other less aggressive antithrombotic regimens. Furthermore, some strategies to minimize the risk of bleeding in this critical group of patients will be briefly described.

## Safety of TAT in patients with AF following DES/PCI

Until recently, there has been little evidence regarding the safety of triple therapy (TAT) in AF patients after PCI or DES. To investigate whether TAT is the optimum antithrombotic strategy in these patients, a number of randomized clinical trials (RCTs) have been conducted. The WOEST trial (What is the Optimal antiplatelet and anticoagulant therapy in patients with oral anticoagulation and coronary stenting) was the first RCT to highlight this issue and it was a tiebreaker between interventionalist and electrophysiologist. While interventionalists advocate for antiplatelet therapy to avoid stents thrombosis, electrophysiologists point to the importance of anticoagulants for stroke prevention in AF, with neither therapy adequate on its own [20]. WOEST compared DAT (warfarin plus clopidogrel 75 mg/day) to TAT (warfarin plus clopidogrel 75 mg/day plus aspirin 80 mg/day) in patients who underwent PCI with stenting. A bleeding event (primary outcome) at 12 months, occurred in 19.4% of the DAT group versus 44.4% of the TAT group (*p *< 0.001). The secondary outcomes [death, MI, stroke, stent thrombosis, or target vessel revascularization (TVR)] appeared in 11.1% of the DAT cohort versus 17.6% of the TAT group (*p *= 0.025) [21]. However, it is worth mentioning that this trial did not compare with NOACs, and it was underpowered to detect differences in stent thrombosis and mortality. The bleeding reported was any bleeding, even if it may not be clinically relevant, which may explain the high bleeding rate in this study. Moreover, there was a low use of proton pump inhibitors (PPIs) at around 30%, which could be another explanation for the high bleeding rate.

Four RCTs compared TAT (with Vit K antagonist) versus DAT (with a P2Y12 inhibitor, mainly clopidogrel) plus multiple adjusted doses of NOACs, rivaroxaban 15 mg o.d. (PIONEER AF-PCI), dabigatran 110 mg or 150 mg b.i.d. (RE-DUAL PCI), apixaban 5 mg b.i.d. (AUGUSTUS), and edoxaban 60 mg o.d. (ENTRUST-AF PCI) in AF patients undergoing PCI (see the summary of these trials in Table 2). The PIONEER AF-PCI randomized 2214 individuals with non-valvular AF who underwent PCI with stent implantation to three treatment groups (approximately 700 each). The study aimed to assess the safety of two different strategies of rivaroxaban (rivaroxaban 10–15 mg daily with P2Y12 inhibitor for 1 year or rivaroxaban 2.5 mg twice daily plus DAPT for 1, 6, or 12 months) and a Vit K antagonist (VKA) strategy (TAT, warfarin with DAPT for 1, 6, or 12 months). Various DAPTs were used, mainly clopidogrel (also prasugrel or ticagrelor) and low-dose aspirin (75–100 mg/day). The primary outcome of clinically relevant bleeding was less in the rivaroxaban population (16.8%, 18.0%; *p *< 0.001) than in the warfarin population (26.7%; *p *< 0.001) at the 1-year follow-up. The diversity of ethnicity was better than in previous trials.; however, the study still lacked good numbers from the East Asian population. This may be important due to the increased incidence of clopidogrel resistance, especially in the Asian population due to the prevalence of genetic polymorphisms [22]. Moreover, this trial lacked robustness in assessing efficacy and secondary outcomes (CV mortality, stent thrombosis, MI, or stroke), and there is inadequacy in the presentation of the clinical adverse events that led to therapy cessation.

**Table 2:** Summary table of RCTs studying DAT versus TAT

Randomized Clinical Trials of Dual versus Triple therapy							
Study	Year	Patients	Blinding	Intervention	Primary outcomes	2nd outcomes	
WOEST	2013	563	Open-label design	Double therapy: Warfarin/VKA + Clopidogrel 75 mg daily Triple therapy: VKA + Clopidogrel 75 mg + ASA 80–100 mg daily	Any bleeding (1 yr): 19.4% versus 44.4% (HR 0.36; 95% CI 0.26–0.50; *P *< 0.0001; NNT 4)	Death, MI, TVR, stroke or stent thrombosis (ST): 11.1% versus 17.6% (HR 0.60; 95% CI 0.38–0.94; *P *= 0.025; NNT 15)	Figure 5
PIONEER AF-PCI	2016	2214	Open-label design	Double therapy: Rivaroxaban 10–15 mg daily + P2Y12 inhibitor for 12 months (*n *= 709) Riva. 2.5 mg BID + DAPT for 1, 6, or 12mo (*n *= 709) Triple therapy: VKA + DAPT × 1,6, or12 mo (*n *= 706)	Clinically relevant bleeding (CRB): 16.8% versus 18.0% versus 26.7%; *P *< 0.001	Death, MI, TVR, stroke, or ST: No significant results of the rate of major adverse CV events (MACE)	Figure 6
RE-DUAL PCI	2017	2725	Open-label design	Double therapy: Dabigatran 110 mg bid + P2Y12 inhibitor (*N *= 981) Dabi. 150 mg bid + P2Y12 inhibitor (*N *= 763) Triple therapy: VKA plus DAPT (*N *= 981)	Major/CRB bleed: Dabi. (110) versus TAT: 15.4% versus 26.9%; *p *< 0.001 Abi. (150) versus TAT: 20.2% versus 25.7%; *p *= 0.002	Death, MI, TVR, stroke, or ST: No significant results of the MACE, with a slight NS increase in the ischemic event in the dabi. arms	Figure 7
ENTRUST-AF-PCI	2019	1506	Open-label design	Double therapy: Edoxaban 60 mg daily plus clopidogrel 75 mg daily for 12 months Triple therapy VKA plus DAPT (clopidogrel 75 mg ASA 100 mg once daily)	Major/CRB bleed: 17% versus 20%;with *p *= 0·001 for non-inferiority (only)	Death, MI, TVR, stroke, or ST: No significant difference in the MACEA and ischemic outcomes	Figure 8
AUGUSTUS	2019	4614	Open-label design	1st randomization: Apixaban 5 mg b.i.d versus VKA 2nd randomization: ASA versus Placebo *4 Groups compared: Api. 5 mg + P2Y12 inhibitor w/o ASA (2#) VKA + P2Y12 inhibitor w/o ASA (2#)	Major bleeding/CRB: 1st random.: 10.5% versus14.7%; *p *< 0.001 2nd random: 16.1% versus 9.0% *p *< 0.001 for non-inferiority only	MACE (death, MI, TVR, stroke or ST) 1st random.: 6.7% versus 7.1% *p *= All NS; except for mortality& hospt 2nd random: 6.5% versus 7.3% *p *= NS, and not tested in the ischemic event	Figure 9

In the RE-DUAL PCI trial (randomized Evaluation of dual antithrombotic therapy plus dabigatran versus TAT with warfarin in Patients with non-valvular AF following PCI), a total of 2725 daily patients with AF who had PCI were randomly allocated (1:1:1) to obtain TAT (warfarin plus a P2Y12 inhibitor and aspirin for a period of 1–3 months), dual antithrombotic therapy (DAT) which includes dabigatran (dose of 110 mg twice daily) plus a P2Y12 inhibitor, or DAT with dabigatran (150 mg twice daily) plus a P2Y12 inhibitor. The most important findings of this study were a significant decrease (11.5%) of major bleeding (clinically meaningful) in DAPT (110 mg dabigatran) when compared with TAT (*p *< 0.001), and 5.5% in DAPT (150 mg dabigatran) when compared with TAT (*p *= 0.002). Furthermore, the safety regarding stent thrombosis between the regimens was almost similar, with just a 1.1% increase in stent thrombosis in the DAT group [23]. Nevertheless, the study had some patients (12%) who received ticagrelor (thienopyridine) regardless of their age or risk of bleeding, which may potentially increase the risk of bleeding associated with ticagrelor, unlike clopidogrel, which is known from other studies to cause less bleeding. Similar to the WOEST, this trial was insufficiently powered to assess the thrombotic event (within a stent), as both DAT regimens showed no significant difference in stent thrombosis when compared to TAT. The definite stent thrombosis in the 110 mg dabigatran group was 1.5% versus 1.3% in the TAT group (HR 1.30 (95% CI 0.63–2.67); *p *= 0.15), while in the 150 mg dabigatran cohort, it was exactly the same as in the TAT group (0.9% vs. 0.9%) (HR = 0.99 (95% CI 0.35–2.81), *p *= 0.98) [23].

The ENTRUST-AF PCI trial enrolled 1506 individuals with AF and recent PCI who were randomized into two groups including TAT (warfarin and clopidogrel 75 mg daily for 1 year and aspirin 100 mg q.d. for 1–12 months) and edoxaban 60 mg daily plus clopidogrel 75 mg daily for 12 months. This trial adds to accumulating evidence that TAT is linked to higher major bleeding rates (20%), compared to 17% in the edoxaban arm (*p *= 0·001 only for non-inferiority), without any significant difference in the ischemic outcomes. This was the first trial to compare DAT with edoxaban against TAT (warfarin-based) in AF patients following PCI [24]. However, this was the only trial that failed to show superiority (*p *= 0.12) of NOAC but only non-inferiority (*p *= 0.001) for bleeding events, compared with a warfarin-based strategy.

**Fig. 3:**
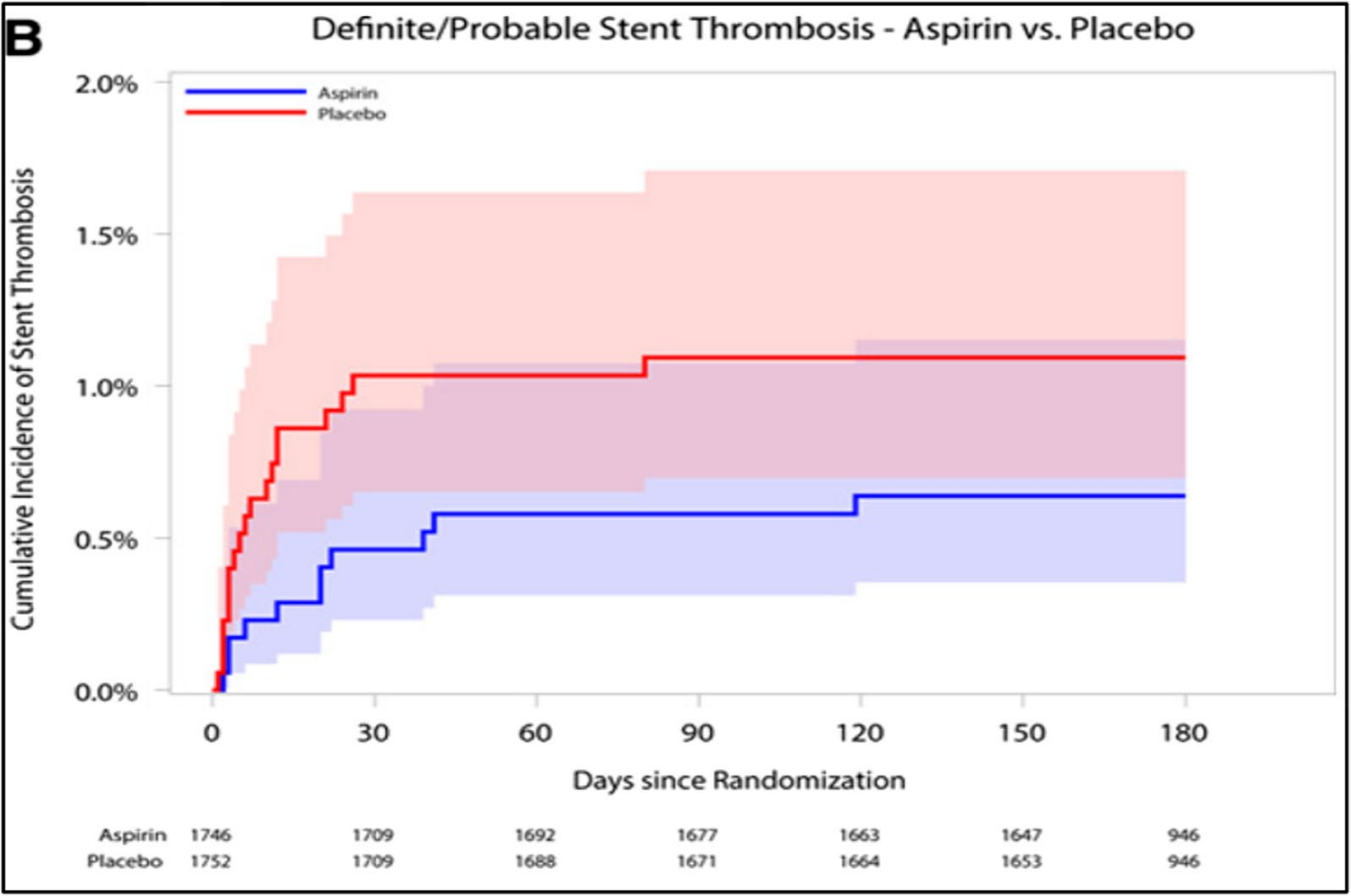
*Stent Thrombosis in Patients With Atrial Fibrillation Undergoing Coronary Stenting in the AUGUSTUS Trial (2020):* As seen in this figure that the incidence of stent thrombosis is largely confined to the first 30 days after stent implantation [26]. 10.1161/CIRCULATIONAHA.119.044584

More recently, the AUGUSTUS trial included 4614 patients to compare two antithrombotic regimens (apixaban vs. VKA/warfarin). In addition, this study compared aspirin against a placebo. This broad objective came after the uncertainty of whether the low rate of bleeding observed on DAT in previous trials was the result of NOAC usage or due to withholding aspirin. Major or clinically significant bleeding occurred in only 10.5% of the individuals who received apixaban, while in 14.7% of those who had warfarin (*p *< 0.001 for non-inferiority and superiority test). Patients who received a placebo had lower bleeding event rates with only 9% as compared with 16.1% of those who received aspirin (*p *< 0.001). For the secondary outcomes, the apixaban group had lower mortalities and hospitalizations than the warfarin group (23.5% vs. 27.4%; *p *= 0.0002), and a comparable ischemic event (including stent thrombosis) in both groups. Regarding the aspirin versus placebo groups, there were similar numbers of deaths, hospitalizations, and ischemic events between the two groups [25]. Interestingly, the AUGUSTUS trial found that most stent thrombosis events occur early within the first month of implantation (Fig. 3), which means that aspirin can still be considered for up to one month in patients at prevailing risk of a thrombotic event [26]. The so-called thrombotic risk is not only driven by the CHADSVASC score but also by other clinical aspects such as the complexity of the performed PCI (number of stents, bifurcation, stent length, etc.) [27]. The trial may be considered a powerful study with reasonable population size to test both primary and secondary outcomes. Since this study is an open-label design, this may lead to selection and observation bias; nevertheless, this seems to be mitigated by blinded outcome assessment.

**Fig. 4:**
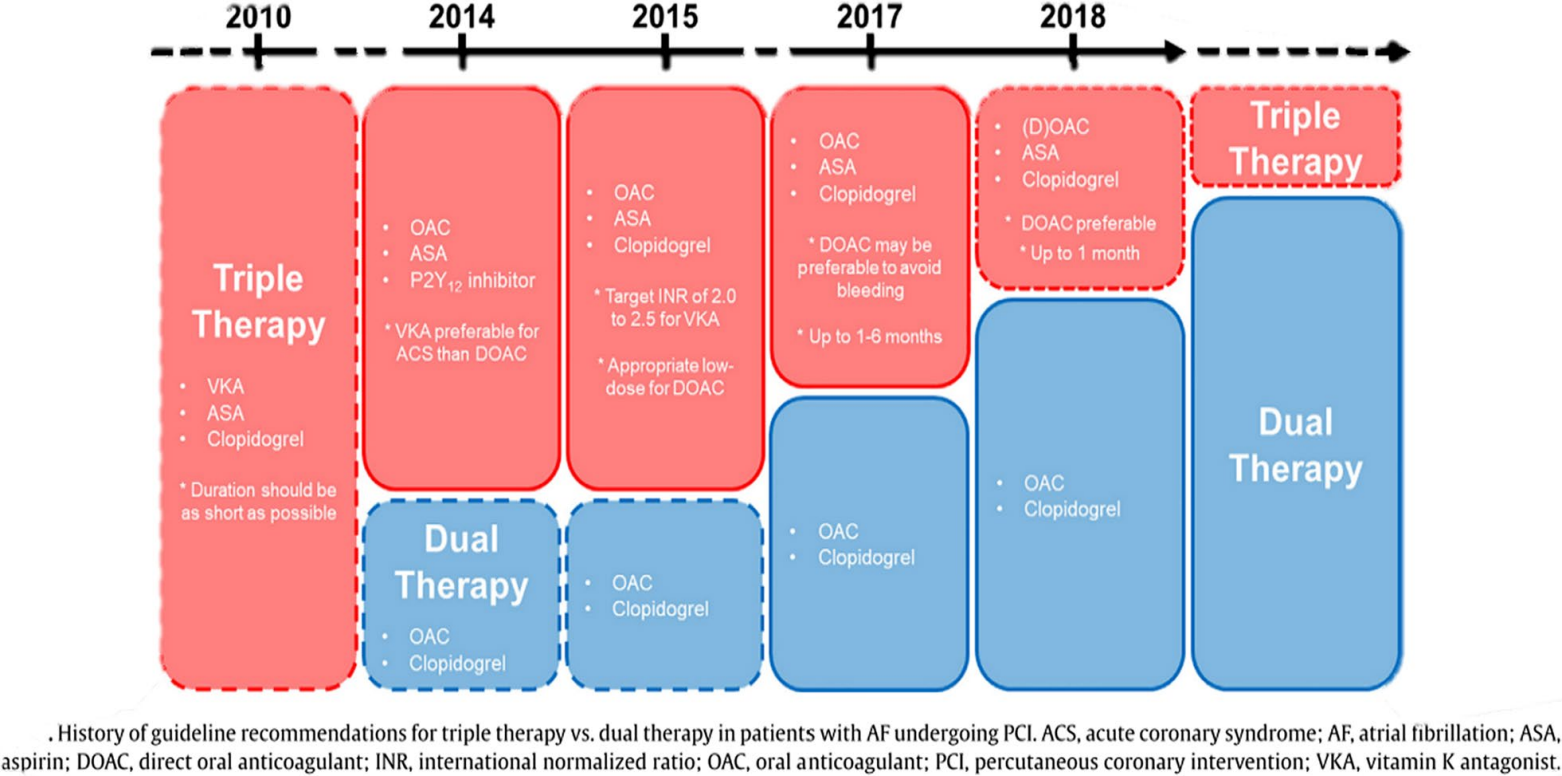
*Triple therapy: A review of antithrombotic treatment for patients with atrial fibrillation undergoing percutaneous coronary intervention*; this diagram shows the history of guidelines and how the use of TAT is becoming less endorsed [28]. DOI: 10.1016/j.jjcc.2018.09.001

**Fig. 5:**
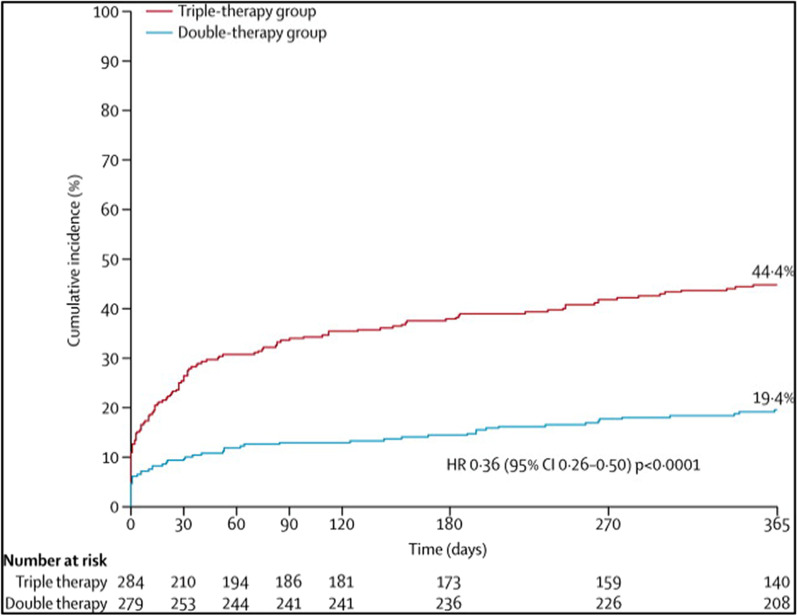
WOEST trial (2013): Primary outcomes of any bleeding, Significant high bleeding in the TAT group compared to DAT [21]. DOI: 10.1016/S0140-6736(12)62177-1

**Fig. 6:**
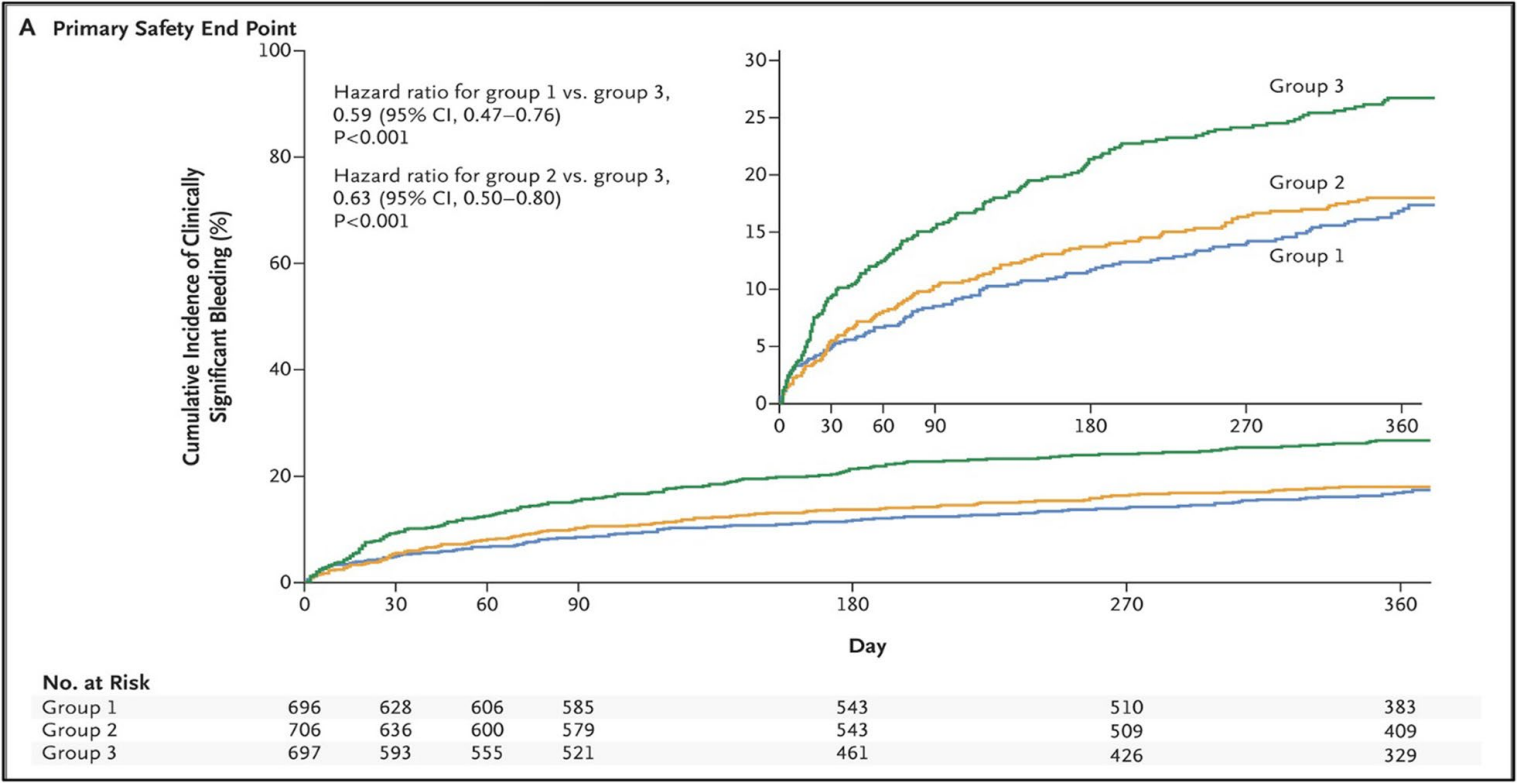
PIONEER AF-PCI (2016) trial: Primary outcomes of major bleeding, Significant high bleeding rates in the TAT group compared to DAT. Group 1 = rivaroxaban 10–15 mg daily + P2Y12 inhibitor, Group 2 = Riva. 2.5 mg BID + DAP, Group 3 = VKA + DAPT [33]. 10.1056/NEJMoa1611594

**Fig. 7:**
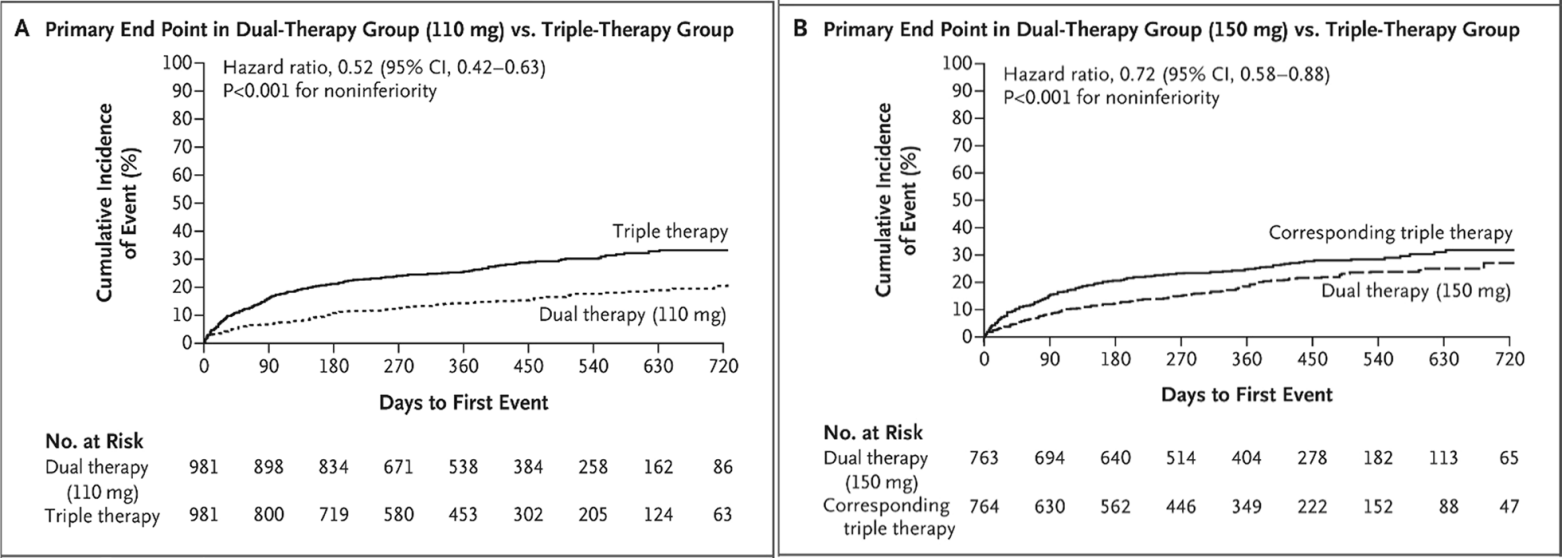
RE-DUAL PCI (2017) trial: Primary outcomes of major bleeding were significantly high bleeding in the TAT group compared to both DAT (dabi. 110 and 150 mg). dabi. 110 mg had a better safety profile (lower bleeding) than TAT and DAT (dabi. 150 mg) [23]. 10.1056/NEJMoa1708454

The accumulating evidence of antithrombotic therapy following PCI in AF patients has been reflected in the guidelines for atrial fibrillation, which resulted in a significant shift from the TAT strategy toward DAT over time (Fig. 4) [28]. Before the publication of the WOEST (2013) and ISAR-TRIPLE (2015) trials, clinical guidelines suggested that these patients should be on TAT (warfarin and DAPT) following PCI. Nowadays, the current ACC/AHA and ESC guidelines recommend the discontinuation of aspirin before the first month (preferably 1 week, depending on the bleeding and thrombotic risk profile) and while continuing NOACs and P2Y12 inhibitors. Due to the bleeding safety profile, the preferred antiplatelet (P2Y12 inhibitor) is clopidogrel, especially in low thrombotic risk patients, which can be discontinued 6–12 months post DES implantation depending on the thrombotic and bleeding risk [29, 30] (Figs. 5, 6, and 7).

**Fig. 8:**
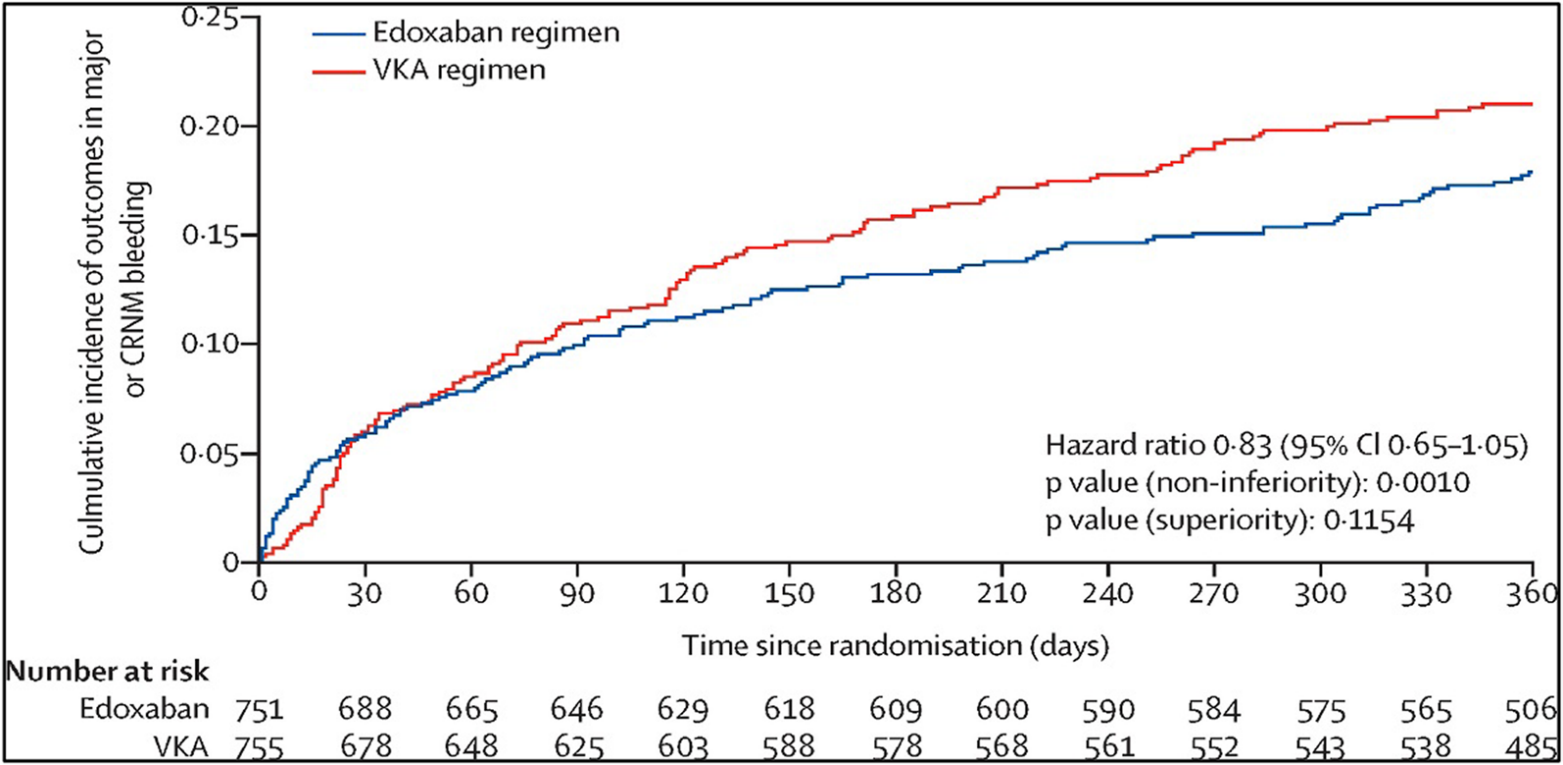
ENTRUST-AF-PCI (2019) trial: Primary outcomes of major bleeding were significantly high in the TAT group compared to both DAT (edoxapan 60 mg). dabi. *failed to show any superiority but only non-inferiority [24]. DOI: 10.1016/S0140-6736(19)31872-0

**Fig. 9:**
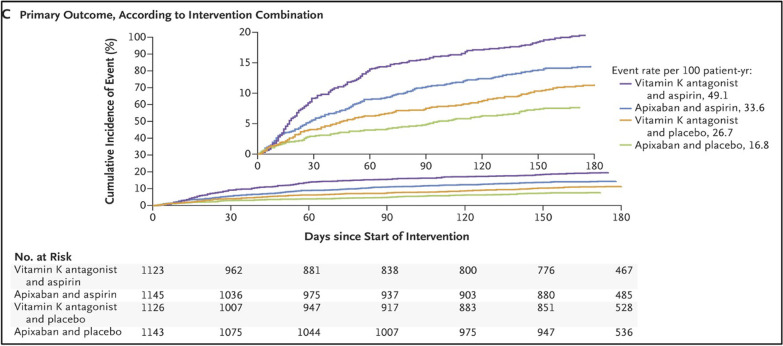
AUGUSTUS trial (2019): Primary outcomes of major/clinically relevant bleeding, showing significant high bleeding in the TAT group compared to DAT. *Note: all patients received P2Y12 inhibitor besides the given medications [25]. 10.1056/NEJMoa1817083

The clinical guidelines and trials are extremely important for identifying the optimal therapy, but they do not tell us everything that happens in the real context of clinical practice. Despite the importance of assessing the safety of triple therapy in these trials, there remain unanswered questions about whether we should consider lower doses of NOACs in addition to antiplatelet therapy, when aspirin is needed due to the high risk of ischemia. Another question is whether the new biodegradable stents would offer better clinical outcomes for these patients. As seen from the AUGUSTUS trial, stent thrombosis is characterized by short-term occurrence (in the first 4 weeks). However, this observation does not fully explain why it is higher during the first month. This leads to the consideration of other possible significant factors such as stent under-expansion and whether intracoronary imaging (e.g., IVUS, OCT) was done to ensure stent optimization would add more clarity to the result. Additionally, some of these trials had a mixed population of ACS patients who were treated by PCI and others who had medical therapy alone. Therefore, further work is required to explore the mechanisms behind early stent thrombosis and to highlight other important clinical questions (Figs. 8 and 9).

There are some strategies cardiologists can use to mitigate bleeding risk when managing such patients. First comes an appropriate assessment of the bleeding and ischemic risk using validated risk scores, such as the CHA2DS2-VASc and the HAS-BLED score. Second is the use of DAT with NOACs and clopidogrel instead of using triple therapy whenever possible [31]. Because of the increased risk of bleeding with ticagrelor and prasugrel, clopidogrel is chosen over the other two antiplatelet agents in stable patients who are already on anticoagulants [32, 33]. Third, in cases of high ischemic risk, clinicians should keep triple therapy as short as possible, and use a low dose of aspirin (< 100 mg daily) [31]. Another important strategy is to consider the routine use of proton pump inhibitors (PPIs) with anticoagulants, which can reduce upper GI bleeding and hospitalization in high-risk patients [32]. Lastly, in critical patients with contraindications to both VKA and NOACs due to high bleeding risk, left atrial appendage occlusion can be an alternative nonpharmacological option to maintain safety and reduce stroke events [4].

## Conclusions

In summary, the complicated relationship between AF, PCI, and antithrombotic therapy poses a significant challenge for clinicians aiming to find an optimal approach that effectively prevents thromboembolic events while minimizing the potential for bleeding complications. As the most frequent sustained arrhythmia that also raises the risk of thrombotic event for patients, making adequate antithrombotic therapy are essential to prevent strokes and other ischemic consequences. The advent of NOACs beyond the traditional warfarin therapy has improved safety profile. The comprehensive analysis of pivotal trials, such as WOEST, PIONEER AF-PCI, RE-DUAL PCI, ENTRUST-AF PCI, and AUGUSTUS, has yielded valuable insights into the relative advantages and disadvantages of various antithrombotic regimens. TAT was once the standard approach following PCI, but the weight of evidence has progressively shifted toward DAT with NOACs and clopidogrel, with the goal of achieving a balance between reducing bleeding risk and maintaining ischemic protection. The disparity in hemorrhage rates and ischemic outcomes between these trials highlights the need for individualized treatment decisions that take into account the unique clinical profiles of individual patients. Guidelines have evolved in response to these findings, guiding clinicians toward refined strategies that favor DAT over TAT, while emphasizing the role of risk assessment scores (CHADSVASC and HAS-BLED) and lowering the duration of aspirin as possible in high bleeding patients, and the use of protective strategies such as proton pump inhibitors to further mitigate bleeding risks. Nevertheless, it is noteworthy that the primary endpoints of these trials have predominantly concentrated on bleeding safety, possibly overshadowing rarer yet critical outcomes such as stent thrombosis, MI, and stroke. Additionally, emerging questions surrounding early stent thrombosis, use of lower NOAC doses need further investigation to enhance therapeutic precision. In this dynamic landscape of AF management following PCI, clinical judgment must align with evidence-based guidance, considering patient characteristics, ischemic risk, and bleeding vulnerability. The synthesis of research, guidelines, and clinical expertise will continue to improve the delicate art of managing anticoagulant strategies that protect against thromboembolic events while maintaining the delicate balance of bleeding risks, toward more tailored therapeutic approaches and improved patient outcomes.

## Data Availability

Not applicable.

## Abbreviations

ACS: Acute coronary syndrome
AF: Atrial fibrillation
PCI: Percutaneous coronary intervention
DAPT: Dual antiplatelet therapy
DAT: Dual antithrombotic therapy
DES: Drug-eluting stent
TAT: Triple antithrombotic therapy
VKA: Vitamin K antagonist
NOACs: Novel oral anticoagulants
DAPT: Dual antiplatelet therapy
MI: Myocardial infarction
ICH: Intracranial cerebral hemorrhage
GI: Gastro-intestinal
CAD: Coronary artery disease
